# Reading Cancer: Chromatin Readers as Druggable Targets for Cancer Treatment

**DOI:** 10.3390/cancers11010061

**Published:** 2019-01-09

**Authors:** Catia Mio, Stefania Bulotta, Diego Russo, Giuseppe Damante

**Affiliations:** 1 Department of Medical Area, University of Udine, 33100 Udine, Italy; giuseppe.damante@uniud.it; 2 Department of Health Sciences, “Magna Graecia” University of Catanzaro, 88100 Catanzaro, Italy; d.russo@unicz.it; 3 Medical Genetics Institute, University Hospital of Udine, 33100 Udine, Italy

**Keywords:** chromatin readers, druggable epigenome, small molecule inhibitors

## Abstract

The epigenetic machinery deputed to control histone post-translational modifications is frequently dysregulated in cancer cells. With epigenetics being naturally reversible, it represents a good target for therapies directed to restore normal gene expression. Since the discovery of Bromodomain and Extra Terminal (BET) inhibitors, a great effort has been spent investigating the effects of chromatin readers’ inhibition, specifically the class of proteins assigned to bind acetylated and methylated residues. So far, focused studies have been produced on epigenetic regulation, dissecting a specific class of epigenetic-related proteins or investigating epigenetic therapy in a specific tumor type. In this review, recent steps toward drug discovery on the different classes of chromatin readers have been outlined, highlighting the pros and cons of current therapeutic approaches.

## 1. Introduction

Mammalian cells can maintain their specific phenotype and adapt to diverse environmental stimuli modifying their epigenome, which is built on a flexible set of mechanisms (creating the epigenetic code), mostly based on a fine cluster of chemical changes interplay, including DNA methylation and histone post-translational modifications (HPTMs) as well as histone variants assembly and 3D chromatin organization [1]. Acting in concert, these mechanisms are responsible for changes in local chromatin structure and dynamics, determining either the compactness or accessibility to specific loci. Moreover, beyond chromatin structure, epigenetic modifications represent a scaffold upon which transcriptional activators/repressors may directly affect gene expression [2]. Chromatin dynamic changes, which include nucleosome unwrapping, re-wrapping, sliding, assembly, and disassembly, involve the formation and/or disruption of interactions within the interfaces between the DNA, histones H3/H4, and H2A/H2B, which are the main components of the nucleosome [3]. Epigenetic modifications constitute a set of tags, which reflect the local state of chromatin; the map of histone covalent modifications is assembled to influence the interactions that stabilize the nucleosome structure by shifting the free energy difference between the fully wrapped nucleosome and altered nucleosome structures [4]. Altogether, these mechanisms build the epigenome, which is sensitive to environmental changes and shapes the flow of information from the genome to the proteome, defining the identity of a cell type [5].

In this review, we will outline recent steps toward drug discovery on the different classes of chromatin readers, highlighting the advantages and challenges in drug screening, focusing on the so-called ‘difficult targets’ and on the advantages of potential treatments.

## 2. Dissecting Chromatin Orthography

Epigenomic reprogramming plays a major role in triggering specific responses from early embryogenesis to the complete development of an organism [6]. Besides DNA methylation, which represents the most well-known reaction to endogenous and environmental stimuli, histone modifications are fundamental for epigenomic reprogramming [7]. Histones may undergo different chemical post-translational modifications (PTMs) to regulate the stable inheritance of cellular memory during mitotic division and fulfill specific regulatory pathways in cell-signaling networks [4]. A discrete number of HPTMs has been discovered, but thus far the main studied ones remain acetylation, methylation, phosphorylation, and ubiquitination. Since the cardinal role of HPTMs both in physiological cell maintenance and in disease states, a wide range of enzymes assigned to their regulation have been discovered and deeply investigated [8]. Three major functional classes of proteins are involved in the regulation and turnover of PTMs: Chromatin writers, which are designated to add a specific PTM; chromatin erasers, which remove them from a certain location within DNA; and chromatin readers, which recognize specific PTMs to accomplish their implicit meaning [4]. The most studied HPTMs and their related enzymes are listed in Table 1.

Relying on the type of HPTMs and their position within the chromatin, histone-modifying enzymes regulate a complex network that can either foster or hinder DNA packaging, in response to specific cellular stimuli [9]. This, in turn, can modify the DNA accessibility to i.e., DNA repair and transcription enzymes, thereby influencing the cellular phenotype without necessarily modifying the genotype. Positively charged histone lysines, which contribute to the tight interaction between DNA and nucleosomes, are among the most frequently modified amino acids: Acetylation of these residues can promote transcription of target genes by modulating the positive–negative charges allowing DNA access, while methylation of specific lysines or arginines has been associated to the opposite effect [10,11]. Undoubtedly, this is not a general statement, since acetylation and methylation of histone tails, depending on both residues and positions, have been related either to transcriptional activation or repression [12].

## 3. Interpreting the “Chromatin Tale”

To activate a specific cellular program, the meaning implied in a peculiar set of histone tags must be disclosed. To this aim, readers display an affinity for specific PTMs and are essential to interpret the rapid interchange between different cellular states. They are usually found in large multi-protein complexes, which include also writers and/or erasers, able to integrate different signaling pathways at the chromatin level [13]. Furthermore, the specificity of a chromatin reader to its cognate PTM is built not only on the direct interaction between the modified residues and the reader’s binding pocket, but also on secondary contact deriving from the flanking histone sequences surrounding the modified residue [14]. For example, a conserved asparagine residue located in the proximity of the bromodomains (BRDs) binding pocket interacts with the acetyl-lysine (Kac) through an anchoring hydrogen bond. Moreover, a conserved tyrosine residue associates the Kac with a water mediated hydrogen bond [15]. Indeed, co-crystal structures with peptidic substrates demonstrated that the Kac is recognized by a central deep hydrophobic cavity, where it is anchored by a hydrogen bond to a fundamental asparagine residue [16]. Modifications in these highly conserved features results in defective BRD-Kac recognition.

**Figure 1:**
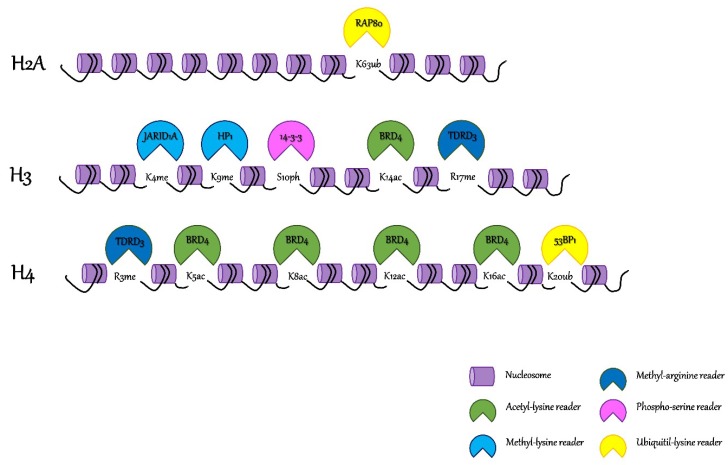
Post-translational modifications (PTMs) on histones tails and their related chromatin readers. Eukaryotic DNA is packaged and subdivided into functional units called nucleosomes (here represented by a purple cylinder). Readers of lysine acetylation (i.e., Bromodomain-containing protein 4 (BRD4)) are represented in green, readers of lysine methylation (i.e., heterochromatin protein 1 (HP1) and Lysine-specific demethylase 5A (JARID1A)) are represented in light blue, readers of arginine methylation (i.e., Tudor domain-containing protein 3 (TDRD3)) are represented in dark blue, readers of lysine ubiquitylation (i.e., Tumor suppressor p53-binding protein 1 (53BP1) and receptor associated protein 80 (RAP80)) are represented in yellow, and readers of serine phosphorylation (i.e., 14-3-3) are represented in pink.

Readers can identify different residues and also diverse modification of the same residue. The complexity is augmented when the same amino acid could undergo several degrees of modification: For example, lysines could exist as mono-methylated, di-methylated, or tri-methylated [13]. For these reasons, chromatin readers are divided into families containing specific binding motifs (Figure 1). Well-characterized examples of reader domains include: Bromodomains (BRDs) typically binding acetyl-lysine; chromatin organization modifier (chromodomains, CRDs), malignant brain tumor (MBT), proline–tryptophan–tryptophan–proline motif (PWWP) as well as Tudor domains generally associated with methyl-lysine, and plant homeodomain (PHD) associated to multiple substrates [17]. Even if 17 types of histone modifications have been assessed, besides acetylation and methylation ones, little is known about the possible reader domains deputed to decipher their role in transcriptional activation and/or repression. Recently, the YEATS domain has been associated to control cell transcription rates by associating with crotonylated lysine residues in active promoters and/or enhancers [18].

The primary readers of N-acetylation of lysine residues is a family of proteins containing an evolutionary conserved domain known as bromodomain. There are more than 40 human proteins containing bromodomains, the most studied one is the Bromodomain and Extra-terminal (BET) family. It comprises four members (BRD2, BRD3, BRD4, and BRDT), which share common structural features with two N-terminal tandem bromodomains. They play a fundamental role in transcriptional elongation and cell-cycle progression, interacting mostly with H4K5/8/12/16ac [19]. Besides BET proteins, BAF180 is a reader protein containing six bromodomains and displaying a distinct pattern of affinity for H3Ksac, which is involved in genome stability, proliferation, and DNA repair [20]. The chemical methylation of lysine residues is engaged by two major families called the Royal Family (accounting for Tudor, Chromo-, and Malignant Brain Tumor (MBT) domains) and the PHD fingers [21]. MBT domains selectively recognize mono and dimethyl-lysine and have been functionally associated with the repression of gene expression while their dysregulation has been linked to several disease states. L3MBTL3, for instance, is found to be deleted in homozygosis in patients with medulloblastoma [22]. Moreover, the Tudor-containing protein 3 (TDRD3) is known to bind methyl-arginine residues and its overexpression has a strong predictive value for poor prognosis in estrogen receptor-negative breast cancers [23].

Phosphorylation is usually linked to transcriptional regulation by extracellular signals, chromatin condensation during mitosis, and DNA damage, and to date, only the 14-3-3 family members and the breast cancer-associated protein carboxy-terminal (BRCT)-containing proteins are known to bind phosphorylated residues on histone tails [24]. Histone ubiquitylation is historically associated to the activation of DNA damage repair (DDR) mechanisms, following mostly double strand brakes (DSBs). The major domains deputed to interact with H2A and H2B ubiquitylated lysines are the ubiquitin-binding domain (UBD) of the UIM-, MIU-, and UBZ-types [25]. RAP80 is an H2AK63 ubiquitylated reader involved in the regulation of DSBs mediated by the homologous recombination (HR) pathway. Specifically, it recruits BRCA1 and other components of the multimeric BRCA1-A complex to the damaged chromatin [26]. Involved in the same pathway, RAD18 promotes HR-mediated DSB repair through the interaction with RAD51C and the SMC5/6 cohesion complex. Since it is scarcely studied, lysines crotonylation has been associated to transcriptional regulation comparable to acetylation. Four YEATS family proteins exist in humans, namely AF9 (encoded by the *MLLT3*—Mixed-Lineage Leukemia Translocated To Chromosome 3—gene), ENL (encoded by the *MLLT1*—Mixed-Lineage Leukemia Translocated To Chromosome 1—gene), glioma amplified sequence 41 (GAS41), and YEATS2/4, which participate with different chromatin-associated complexes involved in transcription elongation, histone modification, and chromatin remodeling [18].

Furthermore, multi-domain protein readers exist, containing two or more different domains through which they can bind different modified residues simultaneously. An example is represented by the Tripartite Motif-containing protein 24 (TRIM24) that can directly associate with chromatin via its tandem PHD-bromodomain modulating gene expression [27]. TRIM24 can bind to euchromatin juxtaposing the two reader domains, creating a single docking site able to interact with H3K23ac through the BRD and to unmethylated H3K4 by the PHD-domain, allowing, for example, estrogen receptor (ER) binding to distal estrogen-response elements [27]. In addition, the complexity of these structures could be extended when different regulatory domains co-exist in the same protein sequence: 53BP1 and RNF168 both contain an ubiquityl-lysine reader and a writer domain able to regulate the damaged site, stimulating either HR or the non-homologous end joining (NHEJ) repair pathways [25].

## 4. Epigenetic Alterations are a Common Place in Cancer

As epigenetic regulation plays a crucial role in normal cellular homeostasis, any variation on this theme could result in abnormal gene expression of key proteins, leading to the onset or the progression of a wide range of diseases, and particularly in cancer [28].

As chromatin constitutes the primary form of protein-DNA assembly in the nucleus, epigenetic modifications can influence all DNA-associated processes, such as transcription, replication, and DNA repair. All these mechanisms are intimately connected to the faithful interpretation and inheritance of the genetic material and therefore are central to inducing and maintaining cell fate choices. Hence, any aberration in this fine-tuned regulation might potentially lead to the accumulation of genomic lesions and ultimately to the loss of cell identity; these consequences are typically associated with the development of neoplasia. Cancer cells, in fact, frequently bear aberrations at readers’ expenses, such as point mutations, translocations, amplifications, and deletions [29]. These alterations can both transform and foster disease progression directly or by affecting gene expression controlled by dysregulated signaling pathways or oncogenic transcription factors [17]. For example, mutations in ING family members (PHD finger containing proteins) have been associated with malignances, such as melanoma and breast cancer [30]. Moreover, the six tandem bromodomain containing protein, *BAF180*, was found to undergo a loss of function mutations in clear cell renal cell carcinoma (CCRCC) [31]. Fusion of *Nucleoporin 98* (*NUP98*) with the PHD containing part of *JARID1A* (or *KDM5A* - *Lysine Demethylase 5A*) drove alterations in leukemia [32]. In NUT-midline carcinoma, a translocation that fuses the *BRD3* or *BRD4* protein to the *NUT* transcriptional regulator creates an oncoprotein that, through binding to acetylated histones, is thought to promote transcription of proliferation genes (e.g., *MYC proto-oncogene*) [33]. As for lysine-crotonylated readers, *GAS41* is an oncogene frequently amplified in human gliomas while *AF9* and *ENL* are two general fusion partners translocated in human mixed lineage leukemia (MLL) [18].

Not only genetic alterations involving chromatin readers occur during neoplastic transformation, but also dysregulation in their expression has been assessed in diverse neoplasms. TRIM24 over-expression correlates with poor survival outcomes in breast cancer patients due to its oncogenic potential in driving ER activity [27]. The YEATS Domain Containing 4 (YEATS4), instead, is found to be overexpressed in several human cancers, such as lung adenocarcinoma, glioblastoma, and colorectal cancer (CRC), promoting cell proliferation through the inhibition of senescence and an increase in multipolar mitotic spindle formation [34]. Despite progresses in the understanding of the molecular events that can drive the epigenetic abnormalities underneath the cancer epigenome, how mammalian cells normally package their genomes for proper gene expression and maintenance of chromosome integrity are questions that remain to be elucidated. Tightly connected is the topic of better characterization of cardinal players in epigenetic programming. As shown in Table 1, most phosphorylation, ADP-ribosylation, and ubiquitylation readers are still elusive, preventing, on one side, a full understanding of the consequences related to HPTMs reading and, on the other side, exploitation of this knowledge for therapeutic purposes.

## 5. Druggable Epigenome

Unlike the genetic mutations, epigenetic changes are naturally reversible and thus catalyzed researchers’ attention as good targets for therapies directed to restore normal gene expression profile, hindering regulators’ dysfunction. Moreover, targeting the epigenome as a viable drug strategy has been fostered and supported by the U.S. Food and Drug Administration (FDA), who, in 2004, granted regular approval to the DNA methyltransferases inhibitor, 5-azacytidine (Vidaza©), for the treatment of all subtypes of myelodysplastic syndrome (MDS) [35]. A couple of years later, Vorinostat (Zolinza©) or suberolyanilidehydroxamic acid (SAHA), a histone deacetylase (HDAC) inhibitor, received FDA approval for the treatment of cutaneous manifestations of cutaneous T-cell lymphoma (CTCL) in patients with progressive, persistent, or recurrent disease [36]. From that instant, epigenetic therapy gained momentum and several chemical agents (epidrugs) mostly targeting chromatin-related enzymes (i.e., writers and erasers) have been developed, entering preclinical studies. Novel and more specific DNMT and HDAC inhibitors were screened in vitro in experimental models of multiple cancer types, such as melanoma, breast cancer, CRC, osteosarcoma, and more. 

EZH2 is a methyltransferase catalyzing H3K27 tri-methylation, an HPTM essential for chromatin compaction and gene silencing [37]. Due to its frequent overexpression and gain-of-function mutations [37,38] in solid cancers and lymphomas, several pharmaceutical companies have embarked on high-throughput screening campaigns, leading to the discovery of small-molecule compounds, inhibiting specifically its methyltransferase activity [39]. EZH2 inhibitors, EPZ-6438 and GSK2816126, for instance, showed anti-neoplastic activity in acute myeloid leukemia (AML) and are currently in clinical trials of lymphoma treatment (Table 2). Moreover, several LSD1 (histone demethylase with specificity toward H3K4 mono/di-methylation) inhibitors are now in clinical trials in refractory AML displaying no *PML-RARα* rearrangement (Table 2).

As inhibition of specific enzymes represents a classical drug developing protocol, more challenging is the inhibition of the binding pocket of a histone-modified reader. Great effort was spent on this subject until researchers’ attention has tremendously been influenced by the discovery of selective inhibitors targeting the BET family of acetyl-lysine readers. Two distinct experimental approaches have led to the development of the first two BET inhibitors (BETis), the benzodiazepine I-BET762 (also known as GSK525762) and the thienodiazepine JQ1, which have been shown to be effective in the downregulation of the *MYC* oncogene in multiple cancer subtypes [29,40]. BET inhibition efficacy is based on the disruption of BET proteins’ interaction with acetylated histones by binding the acetyl-lysine recognition pocket of these chromatin readers. In addition to *MYC* downregulation, BET inhibitors’ biological effects resulted by modulating the expression of cell growth-related proteins. For example, BET inhibition induced a decrease in cell viability that turns out to be specifically related to cell cycle-linked genes’ downregulation (i.e., *MCM5*) in thyroid cancer [41]. Moreover, diverse studies have highlighted how BRD4 is able to regulate DNA damage repair, opening a novel theme on BET-inhibitors related therapeutic approaches [42,43]. BET inhibition results indeed in disproportionately large changes in gene expression, putatively explained by the association of BRD4 with exceptionally large enhancer elements, called “super-enhancers,” which are involved in lineage-specific gene regulation [44].

Overall, BET inhibition interferes with cancer cell cycle progression and DNA repair, hindering tumor progression both in vitro and in vivo. The reversal of cancer cell phenotype (i.e., the promotion of differentiation and growth impairment) because of BET inhibition provided the first proof of concept that readers can act as a potential therapeutic target for cancer treatment [45]. Since JQ1 and GSK525762 development, multiple BETis have been developed showing significant therapeutic effect in a wide range of human diseases [33]. Given that, BET inhibitors have entered clinical trials, proving their anti-neoplastic effects in diverse sub-types of hematological malignancies and solid tumors [46].

A second step in epigenetic readers-based drug discovery has been made by targeting PHD finger-containing proteins that harbor a “reading” specific site for H3K4 methylation. Disulfiram, an acetaldehyde dehydrogenase inhibitor previously FDA-approved in the treatment of alcoholism, and amiodarone-derived compounds were found to inhibit JARID1A PHD finger bound to H3K4me, possibly through physical alterations, causing the ejection of a structural zinc [47,48]. Both compounds showed anticancer effects in preclinical models of AML in which methyl-readers frequently undergo genetic fusions, causing aberrant transactivation of the developmental genes required to maintain the myeloid progenitor state [21,30,47]. However, their potency and selectivity seems poor and ligand-competitive chemicals remain to be developed for these PHD-containing oncoproteins. Additionally, the small molecule, UNC1215, was reported to inhibit L3MBTL3A activity, an MBT containing protein that selectively recognized mono- and di-methyl-lysines, competing with its endogenous targets, such as BCL2 Associated Transcription Factor 1 (BCLAF1), in a comparable way as JQ1 binds to BRD4 [22]. This potent and selective chemical probe represents a promising tool in the field of epigenetic therapy of histone methylation dysregulation, but further knowledge should be developed until UNC1215 may enter human testing.

Furthermore, next generation sequencing (NGS)-based approaches have highlighted how multi-alteration processes underpin tumor development and progression. These high-throughput techniques have great benefits for intensifying the list of putative targets or effectors in drug-based approaches, shedding light on tumor identity and highlighting further therapeutic targets.

## 6. New Challenges

While acetylation readers have been successfully exploited as therapeutic targets in different cancer models, little breakthrough has been made in targeting other chromatin readers. Few recent studies have focused on the dysregulation of YEATS4, known to hinder senescence and to foster cell proliferation, highlighting that its inhibition by means of endogenous or exogenous RNA interference induced apoptosis of CRC cells [34]. These data delineate it as a putative effective target for the clinical treatment of CRC, but, nowadays, no chemical probes targeting YEATS-containing proteins have been synthesized yet. Even if rapid progress has been made in the development of new small-molecule tools targeting the PHD finger domains, selective inhibitors, i.e., targeting TRIM24, frequently overexpressed in breast cancer and associated with poor overall survival and tumor progression, have not yet been developed [48,49]. 

From a therapeutic point of view, BET inhibitors have paved the way for chromatin readers’ inhibition in clinical trials. Over the past decade, BET inhibitors efficacy has been confirmed in several preclinical cancer models, including prostate, breast, colon pancreas, liver, thyroid, and brain carcinoma [33], evaluating a high level of BETi-related cancer sensitivity. To date, 26 clinical trials employing BET inhibitors exist (Table 3), 12 of which are combination trials with hormonal therapy (i.e., fulvestrant, enzalutamide), target-specific antibodies (i.e., anti-PD-L1, anti-JAK2, anti-BCL2), or other epidrugs (i.e., DNA methylation inhibitors).

Combination therapies involving BET inhibitors are a promising therapeutic approach since, besides the biological advantage in multi-target inhibition, they are meant to overcome the impermanent cytotoxic effects proved as single agents [39,46,50]. In fact, despite the promising results collected both in vitro and in vivo, early clinical trials with BET inhibitors have had sundry results, with responses that tend to be short-lived. Side effects included fatigue (50%), thrombocytopenia (29%), decreased appetite (21%), diarrhea (18%), dysgeusia (14%), nausea (14%), neutropenia (11%), and vomiting (11%) [46,51].

Notwithstanding, clinical research on epidrugs is still ongoing, focusing on optimizations for solid tumors treatments, firstly, to fully target the tumor burden in toto. Recently, novel approaches employing carriers, such as multimeric proteins, synthetic or biological nanoparticles, and microbubbles, have shown promising results in preclinical settings [52]. To this aim, HDACi have been encapsulated to enhance tumor targeting hindering side effects, showing an augmented pro-apoptotic effect when combined with radiotherapy [53]. Moreover, combination therapies could benefit from this nanotechnological approach, enclosing multiple small molecules in one nanoparticle targeting cancer cells, reducing side effects coming from single drugs itself, and directing the therapeutic potential right on the tumor burden. To this aim, recently, JQ1 has been inserted in a functionalized nanoparticle together with temozolomide to cross blood-brain barrier and deliver this combination therapy to glioblastoma [54].

**Figure 2:**
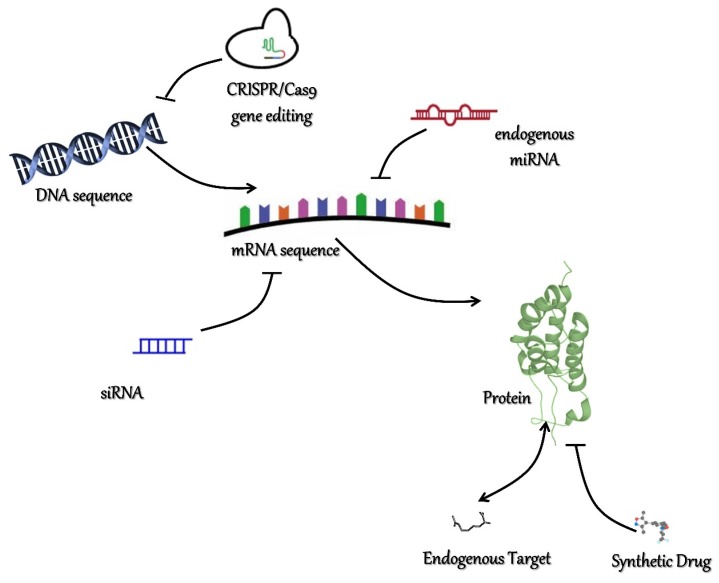
Strategies in chromatin readers’ inhibition. Schematic representation of the diverse methods in readers’ inhibition. Mutations involving readers’ coding sequence or promoters/enhancers’ sequence and causing its mis-regulation could be corrected by the CRISPR/Cas9 gene editing technique. Aberrant gene expression might be suppressed by RNA interference techniques, such as endogenous miRNA targeting or synthetic siRNA delivery. Deregulation could also be hindered at protein levels by means of small molecules acting as exogenous competitors or target mimicking.

Besides epidrugs, epigenetic therapy might rest on small RNA delivery or gene therapy (Figure 2). Adenoviral vectors (AV) used to carry therapeutic genes are common vectors used worldwide within clinical trials and account for most gene therapy-based approaches. AVs are well-tolerated and for decades have been used to induce a local antitumoral immune response; moreover, FDA approved AVs are used in combination with chemotherapy in squamous cell carcinoma of the head and neck (HNSCC) [55]. Possessing mild side effects (i.e., immune system-based reactions, long-term activity), AVs are valuable candidate effects of cancer treatments. The Clustered Regularly Interspaced Short Palindromic Repeats (CRISPR)/Cas9 system has surely revolutionized the field of gene editing and represents a cutting-edge strategy for gene disruption in the host genome [56,57]. Briefly, the CRISPR/Cas9 approach is based on a synthetic fused chimeric single guide RNA (sgRNA) owing a sequence that is designed to be complementary to a target DNA site, introducing or deleting specific sequences or mutations [57,58]. For instance, gene editing with CRISPR/Cas9 could be employed to correct for oncogene deriving from fusion proteins. As an example, *BRD4*-*NUT* translocation is the primary determinant of NMC, in which BRD4 protein to the NUT transcriptional regulator drives the expression of BRD4, promoting transcription of cancer progression-related genes. The aforementioned techniques might aid for the formation of the oncoprotein, hindering tumor expansion.

Besides viral vectors, a huge slice of biotechnological research has produced several polymeric vesicles for delivery within the tumor mass of either targeted drugs or RNA-interference-based approaches (i.e., siRNAs, shRNAs, miRNAs) [59,60,61]. Nowadays, dozens of miRNAs hindering writers, erasers, and readers have been characterized. Indeed, miR-1340 and miR-608 directly suppressed *BRD4* by binding to its coding sequence, reducing in vivo tumor growth in a xenograft mouse model of NMC and hepatocellular carcinoma, respectively [62,63]. MiR-124a inhibited *PHF19* over-expression and hindered cell growth in human glioma [64], while miR-19a and miR-19b down-regulated the expression of *BARD1* in leukemia [65]. Further, synthetic RNA interference-based approaches have been extensively used in preclinical settings to dissect molecular consequences derived from readers’ inhibition, such as PHF19 in melanoma [66], PHF20L1 (Tudor domain-containing protein) in breast cancer [67], and WDR5 in leukemia and pancreatic cancer [68,69], all inhibiting tumor progression in vitro and/or in mouse models.

Nanoparticles are attractive carries because of their non-immunogenic nature and the possibility of being tissue specific with the loading of specific biomarkers on their surface [70,71,72]. All these cutting-edge technologies could be employed to package epidrugs and small RNAs that are not currently usable in vivo, i.e., unstable in plasma, or to prevent systemic side-effects, typical issues in epigenetic therapies. Nonetheless, to date, epigenetic modulation through small molecules (i.e., epidrugs) is by far the most used strategy in clinical workflows, notwithstanding the presence of systemic side effects or little therapeutic benefit in some cancer sub-types.

A deeper understanding of the interconnections between different readers-dependent epigenetic pathways in physiological and pathological conditions will surely help in managing epigenetic therapy.

## 7. Conclusions

Even if a lot of effort has been made, few chemical probes targeting epigenetic readers have been identified to date. Further evaluation must be accomplished to better characterize both readers’ dysfunction in cancer and chemical compounds able to specifically inhibit their functions. Moreover, small molecules targeting epigenetic-related proteins (i.e., writers, eraser and readers) still hold pros and cons. HDAC inhibitors therapy, for instance, lacks in therapeutic effectiveness in ovarian cancer, glioblastoma, and hepatocellular carcinoma [50,73,74]. Although potent as drugs for clinical use, BET inhibitors are not able to singularly bind a bromodomain containing family member, a feature that could certainly reduce therapy-related side effects [13,50].

A step forward in epigenetic therapy has been made with drug combination between epigenetic drugs and chemotherapy (platinum salts) or signaling pathway inhibitors (tyrosine kinases inhibitors or endocrine therapy) [50]. These combination therapies are, nowadays, entering numerous clinical trials, as summarized in Table 3. Surely, better models of the prediction of adverse events are needed to hinder side effects related to epidrugs (either alone or in combination). Another criticism is based on pharmacokinetics (i.e., half-life) that could prevent the use of reader’s inhibitors in vivo. Some small molecules, indeed, possess a short half-life (i.e., JQ1 stability in plasma is about 1 h), impeding their use in clinical trials. Vesicles or carriers could partially solve this issue, allowing drugs to penetrate the tumor burden, releasing the chemical probe within the mass, and avoiding its rapid degradation within the plasma. Moreover, most of the drug screening made so far has been based on focused libraries, but this has been revealed as an unsuccessful strategy for the so-called “difficult targets”, in which the binding domain is shallowed, or crystallography evaluations have classified them as undruggable. A different approach based on small-peptide binging, mirroring an RNA interference approach, may be a better strategy [17,55,59,60]. Ultimately, epidrugs-treated patients sometimes experience resistance or tumor relapses due to epigenetic reprogramming of tumor cells, which no-more rely on the pathway targeted by that epidrug or for the persistence of cancer stem cells (CSCs), hardly embedded within the tumor burden and unattainable by epidrugs. 

In conclusion, investigation leading toward the identification of small-molecules that specifically disrupt dysregulated chromatin-binding proteins, such as those defined by expressed translocations or inappropriate over-expression, could shed light on how chromatin dynamics regulate gene expression. Moreover, dissecting readers’ biology and mechanisms coupled to the newest genomic analysis based on next-generation sequencing could reveal epigenetic addiction underpinning tumorigenic transformation and cancer progression, making precision medicine even closer.

## Figures and Tables

**Table 1:** Summary of the main histone post-translational modifications (HPTMs) and their correlated regulatory enzymes.

Enzymes	Residues	Families	Components
**Methylation**			
Writers	Lysine	Methyltransferases (HMTs)	EZH1/2, MLL1-5, SET1/7/9, SUV39h1/2 SUV40h1/2, G9a, EHMT1, NSD1/2, SMYD2, DOT1L
	Arginine	Methyltransferases (HMTs)	PRMT1/2/4/5/6/7, CARM1
Erasers	Lysine	Demethylases (KDMs)	UTX, JMJD3, KDM1A/B, KDM2A/B, KDM4A/B/C/D, KDM5A/B/C/D, PHF2/8 JHDM1a/b, JHDM2a/b, JMJD2A/B/C/D
	Arginine	Demethylases (RDMs)	JMJD6
Readers	Lysine	Chromodomains Tudor domains PWWP domains Ankyrin repeats	MOF, MRG15 MBT, PHF1/19, TDRD7 BRPF1, NSD1-3 G9a/GLP
	Arginine	Tudor domains	WDR5, TDRD3, SMN1
**Acetylation**			
Writers	Lysine	Acetyltransferases (HATs)	KAT2A/B, KAT3A/B, KAT6/5/7/8, Tip60, CREBBP, EP300, PCAF
Erasers	Lysine	Deacetylases	HDAC1/2/3/4/5/7/8/9/11, SIRT1/2/6/7
Readers	Lysine	Bromodomains	BRD2/3/4/T
**Phosphorylation**			
Writers	Serine	Kinases	CDK1/2, MSK1/2, Mst1, ATR, ATM, RSK2, AMPK, IKK-alpha, AuroraB
	Threonine	Kinases	Haspin/Gsg2, Dlk/Zip
	Tyrosine	Kinases	Mst1, WSTF
Erasers	Serine	Phosphatases	PP2A1, PP1
	Threonine	Phosphatases	PPgamma
	Tyrosine	Phosphatases	EYA1/3
Readers	Serine	14-3-3 proteins BRCT domain	14-3-3β/γ/η/ε/μ
	Threonine	BIR domain	XRCC1, NBS1, BARD1
	Tyrosine	PTB domain	
**Ubiquitynation**			
Writers	Lysine	Ubiquitin-ligases	BRCA1-BARD1, RING1A/RING1B/BMI1
Erasers	Lysine	Isopeptidases	OTUB1/2, BRCC36, USP3/16/26/44
Readers	Lysine		53BP1
**ADP-ribosylation**			
Writers	Glutamate Arginine Glutamate	ADP-ribosyltransferases	PARP1
Erasers	Glutamate Arginine Glutamate	ADP-ribosylhydrolases	PARG, MDO1/2, TARG
Readers	Glutamate Arginine Glutamate	Macrodomains PBZ WWE domain	RNF146 APLF, CHFR

PWWP: Pro-Trp-Trp-Pro; BRCT: BRCA1 C Terminus; BIR: bacuolavirus IAP Repeat; PTB: Phosphotyrosine-binding; PBZ: PAR-binding zinc finger; WWE: Trp-Trp-Glu.

**Table 2:** Some of the clinical trials investigating the antineoplastic effects of chromatin writers/erasers inhibitors.

Target	Intervention	Status	Condition	Study Type	Phase	NCT Number
EZH2	SHR2554	RECRUITING	AML and myelodysplastic syndromes	INTERVENTIONAL	I	NCT03603951
	EPZ-6438	RECRUITING	Advanced Solid Tumors and Hematologic Malignancies	INTERVENTIONAL	I/II	NCT01897571
	CPI-1205	RECRUITING	Advanced Solid Tumors	INTERVENTIONAL	I/II	NCT03525795
DOT1L	EPZ-5676	COMPLETED	AML and myelodysplastic syndromes	INTERVENTIONAL	I	NCT02141828
PRMT5	JNJ-64619178	RECTUITING	Advanced solid tumors	INTERVENTIONAL	I	NCT03573310
	GSK3326595	RECTUITING	Advanced solid tumors	INTERVENTIONAL	I	NCT02783300
LSD1	IMG-7289	ACTIVE	AML and myelodysplastic syndromes	INTERVENTIONAL	I	NCT02842827
	INCB059872	RECRUITING	Advanced Solid Tumors and Hematologic Malignancies	INTERVENTIONAL	I/II	NCT02712905
HDAC	Panobinostat (LBH589)	COMPLETED	HL and MM	INTERVENTIONAL	III	NCT01034163
		ACTIVE	Hematologic Malignancies	INTERVENTIONAL	II	NCT01802879
		COMPLETED	Advanced Solid Tumors and Hematologic Malignancies	INTERVENTIONAL	I	NCT00472368
		COMPLETED	CTCL	INTERVENTIONAL	II/III	NCT00425555
	Belinostat (PXD101)	COMPLETED	AML and myelodysplastic syndromes	INTERVENTIONAL	II	NCT00357032
		COMPLETED	OC	INTERVENTIONAL	II	NCT00301756
	Vorinostat	COMPLETED	Advanced BC		I	NCT00719875
		COMPLETED	Advanced CTCL	INTERVENTIONAL	II	NCT00091559
		ACTIVE	Advanced NSCLC		I	NCT01059552
	CHR-3996	COMPLETED	Advanced Solid Tumors	INTERVENTIONAL	I/II	NCT00697879
	Givinostat	RECRUITING	Chronic myeloproliferative neoplasms	INTERVENTIONAL	II	NCT01761968
	Romidepsin	COMPLETED	T cell lymphoma	INTERVENTIONAL	II	NCT00007345
	KA2507	RECRUITING	Advanced solid tumors		I	NCT03008018
DNMT	SGI-110	COMPLETED	AML and myelodysplastic syndromes	INTERVENTIONAL	I/II	NCT01261312
	Deoxycytidine (Aza TdC)	RECRUITING	Advanced solid tumors	INTERVENTIONAL	I	NCT03366116
	Decitabine	COMPLETED	Metastatic PTC or FTC	INTERVENTIONAL	II	NCT00085293
		COMPLETED	AML and myelodysplastic syndromes	INTERVENTIONAL	II	NCT00492401
	Disulfiram	COMPLETED	PC	INTERVENTIONAL	II	NCT01118741
		RECRUITING	Metastatic BC	INTERVENTIONAL	II	NCT03323346

AML, acute myeloid leukemia; MM; multiple myeloma; HL, Hodgkin’s lymphoma; CTCL, cutaneous T cell lymphoma; OC, ovarian cancer; BC, breast cancer; NSCLC, non-small cell lung cancer; PTC, papillary thyroid cancer; FTC, follicular thyroid cancer; PC, prostate cancer.

**Table 3:** Interventional clinical trials investigating the antineoplastic effects of Bromodomain and Extra Terminal (BET) inhibitors as single agent or in combination with other Food and Drug Administration (FDA)-approved drugs.

BET Inhibitors	Intervention	Status	Condition	Study Type	Phase	NCT Number
I-BET762 (GSK525762)	GSK525762 + FULVESTRANT vs. GSK525762 + PLACEBO	RECRUITING	ER and/or PR-positive/HER2-Negative Advanced or Metastatic Breast Cancer	INTERVENTIONAL	II	NCT02964507
	GSK525762 + ABIRATERONE/ENZALUTAMIDE +PREDNISONE	RECRUITING	Castration-resistant Prostate Cancer	INTERVENTIONAL	I	NCT03150056
	GSK525762 monotherapy	RECRUITING	Relapsed Refractory Hematologic Malignancies	INTERVENTIONAL	I	NCT01943851
	GSK525762 monotherapy	ACTIVE	NUT Midline Carcinoma	INTERVENTIONAL	I	NCT03702036
	MK-8628 monotherapy	COMPLETED	Advanced Solid Tumor	INTERVENTIONAL	I	NCT02259114
	MK-8628 monotherapy	COMPLETED	Hematologic Malignancies	INTERVENTIONAL	I	NCT01713582
	MK-8628 monotherapy	ACTIVE	Hematologic Malignancies	INTERVENTIONAL	I	NCT02698189
FT-1101	FT-1101 + AZACITIDINE vs. FT-1101 + PLACEBO	RECRUITING	Hematologic Malignancies	INTERVENTIONAL	I	NCT02543879
CPI-0610	CPI-0610 + Ruxolitinib vs. CPI-0610 + PLACEBO	RECRUITING	Hematologic Malignancies	INTERVENTIONAL	I-II	NCT02158858
	CPI-0610 monotherapy	COMPLETED	Multiple Myeloma	INTERVENTIONAL	I	NCT02157636
	CPI-0610 monotherapy	ACTIVE	Lymphoma	INTERVENTIONAL	I	NCT01949883
INCB054329	INCB054329 monotherapy	COMPLETED	Advanced Solid Tumors and Hematologic Malignancies	INTERVENTIONAL	I-II	NCT02431260
RO6870810	RO6870810 + Atezolizumab vs. RO6870810 + PLACEBO	RECRUITING	Advanced Ovarian Cancer and TNBC	INTERVENTIONAL	I	NCT03292172
	RO6870810 and VENETOCLAX + RITUXIMAB vs. RO6870810 and VENETOCLAX + PLACEBO	RECRUITING	DLBCL	INTERVENTIONAL	I	NCT03255096
GSK2820151	GSK2820151 monotherapy	ACTIVE	Advanced or Recurrent Solid Tumors	INTERVENTIONAL	I	NCT02630251
ZEN003694	ZEN003694 monotherapy	COMPLETED	Metastatic Castration-resistant Prostate Cancer	INTERVENTIONAL	I	NCT02705469
	ZEN003694 + ENZALUTAMIDE vs. ZEN003694 + PLACEBO	RECRUITING	Metastatic Castration-resistant Prostate Cancer	INTERVENTIONAL	I-II	NCT02711956
BMS-986158	BMS-986158 and NIVOLUMAB	RECRUITING	Advanced Tumors	INTERVENTIONAL	I-II	NCT02419417
ABBV-075	ABBV-075 and VENETOCLAX	RECRUITING	Solid Tumors	INTERVENTIONAL	I	NCT02391480
GS-5829	GS-5829 + ENZALUTAMIDE vs. GS-5829 + PLACEBO	ACTIVE	Metastatic Castration-resistant Prostate Cancer	INTERVENTIONAL	I-II	NCT02607228
	GS-5829 + FULVESTRANT vs. GS-5829 + EXEMESTANE	COMPLETED	Advanced Solid Tumors and Lymphomas	INTERVENTIONAL	I	NCT02392611
PLX51107	PLX51107 monotherapy	RECRUITING	Advanced Solid Tumors and Hematologic Malignancies	INTERVENTIONAL	I	NCT02683395

FULVESTRANT, anti-estrogen receptor; ABIROTERONE, ENZALUTAMIDE, anti-androgen; PREDNISONE, corticosteroid; AZACITADINE, DNA methylation inhibitor; RUXOLITINIB, JAK2 inhibitor; ATEZOLIZUMAB, anti-PD-L1 monoclonal antibody; VENETOCLAX, BCL2 inhibitor; RITUXIMAB, anti-CD20 monoclonal antibody; NIVOLUMAB, anti-PD1 monoclonal antibody; EXEMESTONE, anti-estrogen. BC, breast cancer; CR-PC, castration-resistant prostate cancer; NMC, NUT midline carcinoma; NSCLC, non-small cell lung cancer; TNBC, triple negative breast cancer; GBM, glioblastoma multiforme; MM; multiple myeloma; OC, ovarian cancer; DLBCL, diffuse large B-cell lymphoma.
